# Deep learning-assisted detection and segmentation of intracranial hemorrhage in noncontrast computed tomography scans of acute stroke patients: a systematic review and meta-analysis

**DOI:** 10.1097/JS9.0000000000001266

**Published:** 2024-03-15

**Authors:** Ping Hu, Tengfeng Yan, Bing Xiao, Hongxin Shu, Yilei Sheng, Yanze Wu, Lei Shu, Shigang Lv, Minhua Ye, Yanyan Gong, Miaojing Wu, Xingen Zhu

**Affiliations:** a Department of Neurosurgery, The Second Affiliated Hospital, Jiangxi Medical College, Nanchang University; b Jiangxi Key Laboratory of Neurological Tumors and Cerebrovascular Diseases; c Jiangxi Health Commission Key Laboratory of Neurological Medicine; d Institute of Neuroscience, Nanchang University, Nanchang, Jiangxi, People’s Republic of China

**Keywords:** deep learning, hematoma segmentation, intracerebral hemorrhage, volume quantification, systemic review

## Abstract

**Background:**

Deep learning (DL)-assisted detection and segmentation of intracranial hemorrhage stroke in noncontrast computed tomography (NCCT) scans are well-established, but evidence on this topic is lacking.

**Materials and methods:**

PubMed and Embase databases were searched from their inception to November 2023 to identify related studies. The primary outcomes included sensitivity, specificity, and the Dice Similarity Coefficient (DSC); while the secondary outcomes were positive predictive value (PPV), negative predictive value (NPV), precision, area under the receiver operating characteristic curve (AUROC), processing time, and volume of bleeding. Random-effect model and bivariate model were used to pooled independent effect size and diagnostic meta-analysis data, respectively.

**Results:**

A total of 36 original studies were included in this meta-analysis. Pooled results indicated that DL technologies have a comparable performance in intracranial hemorrhage detection and segmentation with high values of sensitivity (0.89, 95% CI: 0.88–0.90), specificity (0.91, 95% CI: 0.89–0.93), AUROC (0.94, 95% CI: 0.93–0.95), PPV (0.92, 95% CI: 0.91–0.93), NPV (0.94, 95% CI: 0.91–0.96), precision (0.83, 95% CI: 0.77–0.90), DSC (0.84, 95% CI: 0.82–0.87). There is no significant difference between manual labeling and DL technologies in hemorrhage quantification (MD 0.08, 95% CI: −5.45–5.60, *P*=0.98), but the latter takes less process time than manual labeling (WMD 2.26, 95% CI: 1.96–2.56, *P*=0.001).

**Conclusion:**

This systematic review has identified a range of DL algorithms that the performance was comparable to experienced clinicians in hemorrhage lesions identification, segmentation, and quantification but with greater efficiency and reduced cost. It is highly emphasized that multicenter randomized controlled clinical trials will be needed to validate the performance of these tools in the future, paving the way for fast and efficient decision-making during clinical procedure in patients with acute hemorrhagic stroke.

## Introduction

Highlights

- Deep learning (DL) technologies can accurately detection intracranial hemorrhage (ICH).
- DL technologies have comparable performance in ICH segmentation and quantification to experienced radiologists or clinicians.
- DL techniques take less time to process images than manual labeling.

China is one of the countries grappling with the highest incidence and mortality rates of stroke worldwide^1,2^. The staggering numbers of 247 cases per 100 000 person-years for stroke incidence and 115 cases per 100 000 person-years^1^ for stroke mortality underscore the severity of the situation when compared to the global average incidence of 85 to 117 cases^3^ and mortality of 30 to 114 cases per 100 000 person-years^2^. Additionally, it has been observed that the proportion of intracranial hemorrhage (ICH) stroke in China, accounting for 25%, is significantly higher than in high-income countries (9–13%)^3^ and comparable to other low-income countries to middle-income countries (14–27%)^3^. The type of acute hemorrhage stroke can be divided into intraparenchymal hemorrhage (IPH), intraventricular hemorrhage (IVH), subdural hemorrhage (SDH), epidural hemorrhage (EDH), and subarachnoid hemorrhage (SAH)^4^. The volume of hematoma plays a crucial role in determining the mortality and long-term neurological outcome for patients with ICH^5,6^. It is of utmost importance in evaluating the progression of the disease, devising targeted interventions and strategies, and managing rehabilitation in the early stages of ICH. Therefore, accurate and rapid detection and segmentation of hemorrhage lesions are imperative for effective medical intervention and optimal patient care.

Noncontrast computed tomography (NCCT) is a standard imaging modality for ICH due to its fast speed of image acquisition and strong contrast between hematoma and healthy brain tissue. It also provides detailed information on the bleeding subtype, location, shape, and volume^7^. Application of ABC/2 formula in NCCT scans is commonly used to describe the subtype of hemorrhage and calculate the hematoma volume^8^, but it is prone to errors in volume quantification of irregular hematomas or bleeding at special sites. Moreover, the large number of interpretations on NCCT scans increase the burden of radiologists and make a misdiagnosis of ICH stroke. Therefore, it is an urgent need for an efficient technology that can accurately detect and segment the suspicious hemorrhage lesions.

As a branch of artificial intelligence (AI), deep learning (DL) has shown great promise in the field of medical imaging analysis^9^. DL-based bleeding detection and segmentation of ICH based on NCCT scans has gradually become a commercial technology to assist clinicians in decision-making, prognosis prediction, and the development of precision medicine^5,10–17^. Previous studies have focused on the early detection of ICH^12,13,18^, and applications of DL algorithms for ICH segmentation were well-established. However, there is no systematic evidence that proving DL technology can assist clinicians in automatically detect and segment the hemorrhage lesions, and whether it can benefit for decision-making during clinical procedures.

The authors hypothesized that DL technologies can yield a comparable performance in ICH detection and quantification to that of experienced radiologists and clinicians. To verify this hypothesis, we conducted a novel systemic review and meta-analysis based on recent research, and revealed the performance of DL technologies in ICH detection and segmentation in acute stroke patients.

## Methods

This systemic review was structured and written according to Preferred Reporting Items for Systematic Reviews and Meta-Analyses’ extension for diagnostic accuracy studies statement (PRISMA-DTA, Supplemental Digital Content 1, http://links.lww.com/JS9/C81, Supplemental Digital Content 2, http://links.lww.com/JS9/C82)^19^ AMSTAR 2 (Supplemental Digital Content 3, http://links.lww.com/JS9/C83)^20^.

### Search strategy and selection criteria

To identify all potentially eligible studies, the literature search was conducted in two public databases (Medline (via PubMed) and EMBASE) from inception to November 2023 by using the predetermine keywords: ((‘Deep learning’[MeSH Terms] OR ‘Machine Learning’[MeSH Terms] OR ‘Artificial Intelligence’[MeSH Terms] OR ‘Algorithms’[MeSH Terms]) AND (‘hemorrhagic stroke’[Title/Abstract] OR ‘hemorrhagic’[Title/Abstract] OR ‘hemorrhagic stroke’[MeSH Terms] OR ‘Intracerebral hemorrhage’[Title/Abstract] OR ‘stroke’[MeSH Terms] OR ‘cerebral hemorrhage’[Title/Abstract] OR ‘Cerebrovascular Disorders’[MeSH Terms]) AND (‘segment*’[Title/Abstract] OR ‘Detection’[Title/Abstract]) AND ‘English’[Language]) NOT ‘review’[Publication Type]. To perform a comprehensive search, two reviewers manually screened previous reviews and reference lists of relevant studies to obtain eligible studies.

The inclusion criteria are as follows: (1) Studies reported accuracy metrics for DL algorithms in ICH detection using NCCT scans; (2) Studies reported segmentation or volume quantification performance of ICH using DL algorithms. Any of the following studies were excluded: (1) Studies did not provide available data leading to unable to perform meta-analysis; (2) case report, case series, review; (3) Studies written in non-English text. No restriction was imposed on the geographic region or the year of publication. After searching, all hits will be downloaded from databases and imported to EndNote. Firstly, we removed the duplicates. Next, two reviewers will independently evaluate the remaining articles at the titles and abstracts level, and then at the full text level to assess their eligibility according to predetermined inclusion and exclusion criteria. Any dispute in this process will be arbitrated by the third reviewer.

### Data extraction and interest outcomes

Two reviewers independently recorded the following information for each included studies by using a standardized Excel file: author name, year of publication, country, ICH subtypes classification, model, population, test set, type of internal validation, whether or not perform an external validation, DL task, ground truth definer, and performance measurements. All performance measurements were extracted from test dataset. Any dispute was settled down by the third reviewer. If necessary, to address missing data, an e-mail was sent to corresponding author.

The objective of this systemic review was to evaluate the performance of DL algorithms for the detection and segmentation of all ICH subtypes. Hence, the primary outcomes were sensitivity, specificity, and dice similarity coefficient (DSC); while the second outcomes were positive predictive value (PPV), negative predictive value (NPV), precision, area under receiver operation characteristic curve (AUROC), process time, and volume of bleeding. The diagnosis metrics were divided into four categories: true positive (TP), true negative (TN), false positive (FP), and false negative (FN). Sensitivity, specificity, DSC, precision, PPV, NPV can be calculated using the following equations:


$$S\mathrm{ensitivity}=\frac{\mathrm{TP}}{\mathrm{TP}+\mathrm{FN}}$$

$$\mathrm{Specificity}=\frac{\mathrm{TN}}{\mathrm{TN}+\mathrm{FP}}$$

$$\mathrm{Precision}=\frac{\mathrm{TP}}{\mathrm{TP}+\mathrm{FP}}$$

$$\mathrm{DSC}=\frac{2\times \mathrm{TP}}{2\times \mathrm{TP}+\mathrm{FN}+\mathrm{FP}}$$

$$\mathrm{PPV}=\frac{\mathrm{TP}}{\mathrm{TP}+\mathrm{FP}}$$

$$\mathrm{NPV}=\frac{\mathrm{TN}}{\mathrm{TN}+\mathrm{FN}}$$

### Bias assessment

The systematic review included diagnostic studies designed to assess the accuracy of DL algorithms in diagnosing and quantifying ICH. To assess the quality of methodological for the included studies, the Quality Assessment of Diagnostic Accuracy Studies (QUADAS-AI) tool was applied^21^. The tool consists of four main parts: patient selection, index test, reference standard, flow, and timing. Each included studies were independently assessed by two reviewers, and any controversy was judged by the third reviewer.

### Statistical analysis

For diagnostic meta-analysis, we employed bivariate model to compute summary estimates of sensitivity, specificity, and AUC^22^. Other independent proportion and their differences were calculated and merged via DerSimonian and Laird random-effects method^23^. Firstly, we pooled all available data, including independent proportion data and raw diagnostic accuracy data. Secondly, where raw diagnostic accuracy data were available, we utilized SROC model to evaluate the relationship between sensitivity and specificity^24^.

Where studies reported TP, TN, FP, FN, SROC model was used to pooled results. We conducted a Fagan’s line diagram to explore the relationship between pretest probability, the likelihood ratio (LR), and post-test probability. Furthermore, an LR dot plot served as a tool for evaluating the clinical utility of a diagnostic approach. The LR plot was segmented into four quadrants, with the top-left quadrant meaning DL model owning both confirmatory and exclusionary diagnostic ability, the top-right quadrant meaning confirmatory ability, the bottom-left quadrant representing exclusionary diagnostic ability, and the bottom-right quadrant indicating that the DL model lacked both diagnostic and exclusionary ability. The assessment of the test’s clinical significance is based on the location of the combined effect size on the plot. The Deek’s funnel plot was used to determine whether there was publication bias in the included studies; the closer the angle between the regression line and the vertical axis in the Deek’s funnel plot was to 90°, the less likely publication bias was present; when *P*>0.05, there was no publication bias. Above statistical treatment was performed with Stata 17 midas package.

For compared meta-analysis, we calculated odds ratio (OR) and mean difference (MD)/weight mean difference (WMD) with corresponding 95% CIs to estimate binary variable and continuous variable, respectively. We adopt Tufanaru C method to determine synthesis model^25^: when less than five studies, fixed-effect model (Inverse variance method) is selected to combine data. When included studies more than 5, we obey the following regulations to determine synthesis model: random-effects model (Der Simonian and Laired method) is selected in case of *I*^2^ more than 50%; while, a fixed-effect model (Inverse variance method) is selected when *I*^2^ less than 50%.

Study-specific estimates with its 95% CIs were computed and represented on forest plots. Heterogeneity between studies was assessed using the Cochran Q statistic (*χ*^2^ test), and *I*^2^ values were used to describe the level of interstudy heterogeneity (25–50% was considered to be low heterogeneity, 51–75% was moderate, and >75% was high heterogeneity). Funnel plots were used to visualize publication bias, and furthermore, *P*-values of the Egger regression test were computed to estimate publication bias, with a *P*-value <0.05 deemed as significant bias^26^.

For above analysis, beyond a *P*-value of <0.10 for *χ*^2^ test, a *P* value of <0.05 is deemed as statistical significance. All statistical treatment was conducted with Stata V.17.0 software (Stata Corp LP).

## Results

### Description of included studies

**Figure 1:**
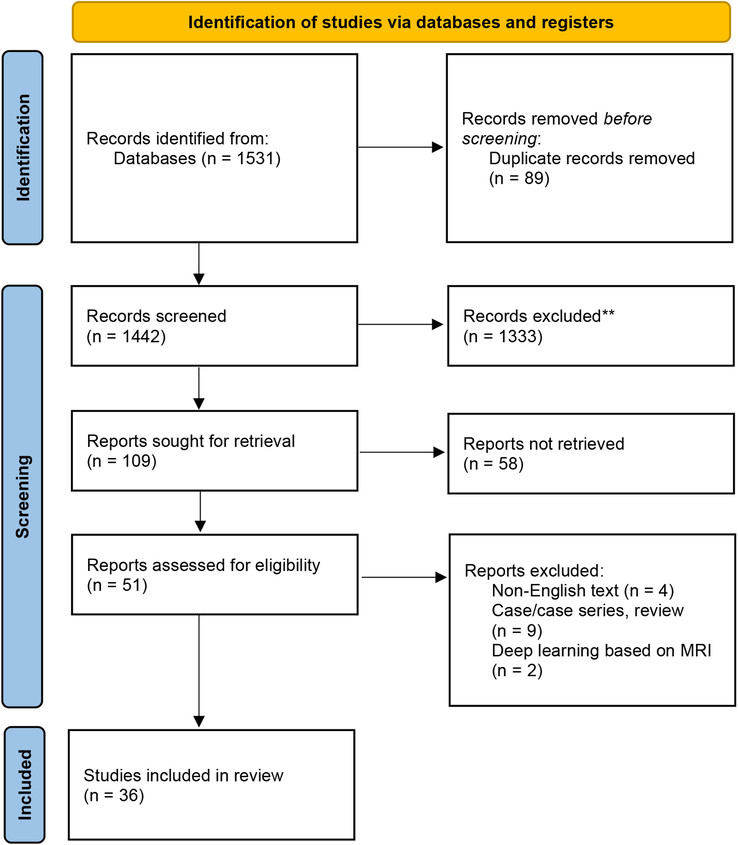
Flowchart of the study selection process.

The search identified 1531 hits from two databases and, finally, 36 studies were included in this systematic review (Fig. 1). The included 36 studies^15,27–61^ were published from 2012 to 2023. USA (*n*=14) and China (*n*=12) were two most countries with the largest number of publications. Nine included studies^27,28,31,35,40,41,55,60^ run internal validation via cross-validation method, 14 included studies^29,30,32–34,37–39,46,47,56,57,59,61^ via random split method, 3 included studies^42,48,50^ via hold-out method, and 10 included studies^15,36,43,45,49,51–54,58^ not report or not application. Twelve included studies^30,32,33,37,39,41,46,47,50,51,55,56,61^ performed a further external validation. Regarding the target tasks for processing the NCCT imaging, 30 studies^15,28,30–40,43–47,50–61^ focused on detection, 27 studies^15,27,29,31,33–35,38–45,48–53,56–61^ focused on segmentation, and 18 studies^15,29,31,33,35,38–40,43–45,50,52,56–59,61^ focused on volume quantification. Detailed information of included studies was presented in Supplementary Table 1 (Supplemental Digital Content 4, http://links.lww.com/JS9/C84).

**Figure 2:**
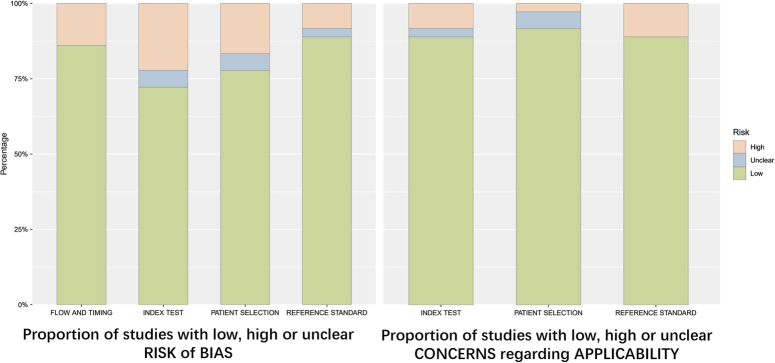
Summary of overall quality assessment for included studies. Green means ‘low risk’; Blue ‘means unclear’; Orange means ‘high risk’.

Results of the quality assessment of all included studies were presented in Figure 2. Quality assessment for individual studies can be found in Supplementary Table 2 (Supplemental Digital Content 4, http://links.lww.com/JS9/C84). In summary, nine studies^28,32,33,39,40,46,52,56,61^ had low risk of bias and applicability in all domains; while, the rest studies had risk of bias in at least one domain.

### Pooled results

The summary pooled results can be found in Table 1.

**Table 1:** Summary estimates of pooled outcomes.

Outcomes	Included studies	Estimate	95% CI	*P* value	*I*^2^ (%)
Sensitivity	25	0.89	(0.88–0.90)	–	99.81
Specificity	18	0.91	(0.89–0.93)	–	99.95
AUROC	6	0.94	(0.93–0.95)	–	99.88
PPV	10	0.92	(0.91–0.93)	–	99.83
NPV	6	0.94	(0.91–0.96)	–	99.91
Precision	9	0.83	(0.77–0.90)	–	99.99
DCS	20	0.84	(0.82–0.87)	–	99.98
Volume of bleeding (Manual vs. DL)	2	MD=0.08	(−5.45–5.60)	0.98	0
Process time (Manual vs. DL)	2	**WMD=2.26**	**(1.96**–**2.56)**	**0.001**	87.67

AUROC, area under receiver operation characteristic curve; DL, deep learning; MD, mean difference; NPV, negative predictive value; PPV, positive predictive value; WMD, weight mean difference.

Bold indicates *P*<0.05.

### Detection performance

Sensitivity, specificity, AUROC, PPV, NPV, and precision were calculated to assess DL models detection performance. twenty-five studies^28,30–34,36–38,43,45–48,51–61^ recorded sensitivity (Supplementary Figure 1, Supplemental Digital Content 4, http://links.lww.com/JS9/C84, pooled sensitivity 0.89, 95% CI: 0.88–0.90), 18 studies^28,30,32–34,36–38,43,46–48,51–56^ recorded specificity (Supplementary Figure 2, Supplemental Digital Content 4, http://links.lww.com/JS9/C84, pooled specificity 0.91, 95% CI: 0.89–0.93), six studies^28,30,32,37,46,52^ recorded AUROC (Supplementary Figure 3, Supplemental Digital Content 4, http://links.lww.com/JS9/C84, pooled AUROC 0.94, 95% CI: 0.93–0.95), 10 studies^31,33,36–38,51,54,57–59^ reported PPV (Supplementary Figure 4, Supplemental Digital Content 4, http://links.lww.com/JS9/C84, pooled PPV 0.92, 95% CI: 0.91–0.93), six studies^33,36–38,51,54^ reported NPV (Supplementary Figure 5, Supplemental Digital Content 4, http://links.lww.com/JS9/C84, pooled NPV 0.94, 95% CI: 0.91–0.96), nine studies^28,29,31,35,39,53,55,56,61^ reported precision (Supplementary Figure 6, Supplemental Digital Content 4, http://links.lww.com/JS9/C84, pooled precision 0.83, 95% CI: 0.77–0.90).

**Figure 3:**
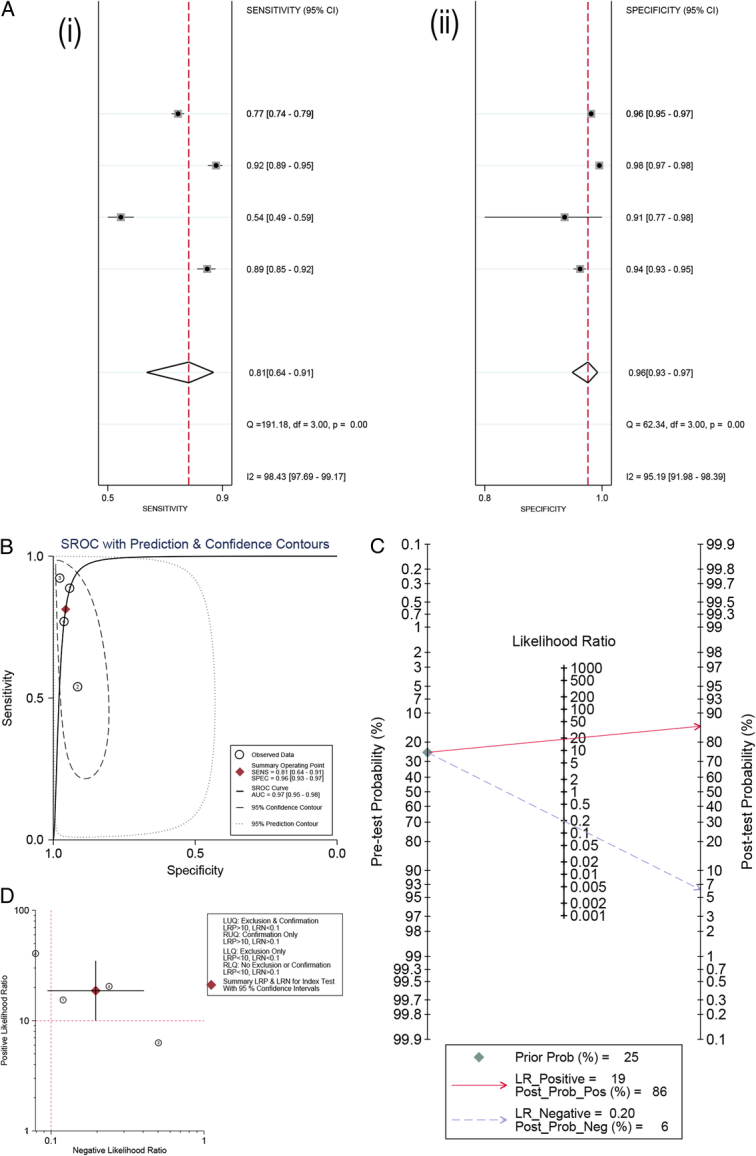
Results of raw diagnostic accuracy data: (Ai) Forest plots of pooled sensitivity (Aii) Forest plots of pooled specificity; (B) SROC showed average sensitivity and specificity estimate of the study results with 95% confidence region. The 95% prediction region represents the confidence region for a forecast of the true sensitivity and specificity in a future study; (C) Fagan’s line diagram; (D) LR dot plot. LLQ, left lower quadrant; LRN, likelihood ratio negative; LRP, likelihood ratio positive; LUQ, left upper quadrant; RLQ, right lower quadrant; RUQ, right upper quadrant; SROC, summary receiver operating characteristic.

Four studies^34,36,48,54^ provided raw data of the DL models for detection ICH. A SROC model was employed to evaluate the relationship between sensitivity and specificity. Pooled sensitivity (Fig. 3Ai), specificity (Fig. 3Aii), and AUROC (Fig. 3B) were 0.81 (95% CI: 0.64–0.91), 0.96 (95% CI: 0.93–0.97) and 0.97 (95% CI: 0.95–0.98), respectively. We conducted a Fagan’s line diagram (Fig. 3C) to explore the relationship between pretest probability, the likelihood ratio (LR), and post-test probability. It can be seen from the diagram the likelihood ratio positive (PLR) of the upper slash was 19%, the pretest probability was set at 25%, and the post-test probability was 86%; the likelihood ratio negative (NLR) of the lower slash was 0.20, the pretest probability was set at 25%, and the post-test probability was 6%. This meant that for the cases included in this meta-analysis, DL model had ability to accurately diagnose 86% of the patients when DL model showed positive results; on the contrary, when DL model showed negative results, only 6% were likely to be misdiagnosed by DL. Fagan’s line diagram suggested that DL model own excellent clinical applicability for the detection of ICH. The LR dot plot revealed that the combined value fell within the upper right quadrant, specifically with LRP >10 and LRN >0.1 (Fig. 3D). This signified that the DL model exhibited confirmatory capabilities for identifying ICH.

### Segmentation performance

Dice similarity coefficient was commonly used to assess the similarity of the two different samples. Here, we employed DSC to express DL model segmentation performance with value ranged from 0 to 1. A total of 20 studies^27,29,34,35,39–45,48–50,56–61^ presented DSC and pooled data reached 0.84 (95% CI: 0.82–0.87) (Supplementary Figure 7, Supplemental Digital Content 4, http://links.lww.com/JS9/C84), which suggested that DL model had good segmentation property for ICH in NCCT images.

### Volume quantification performance

**Figure 4:**
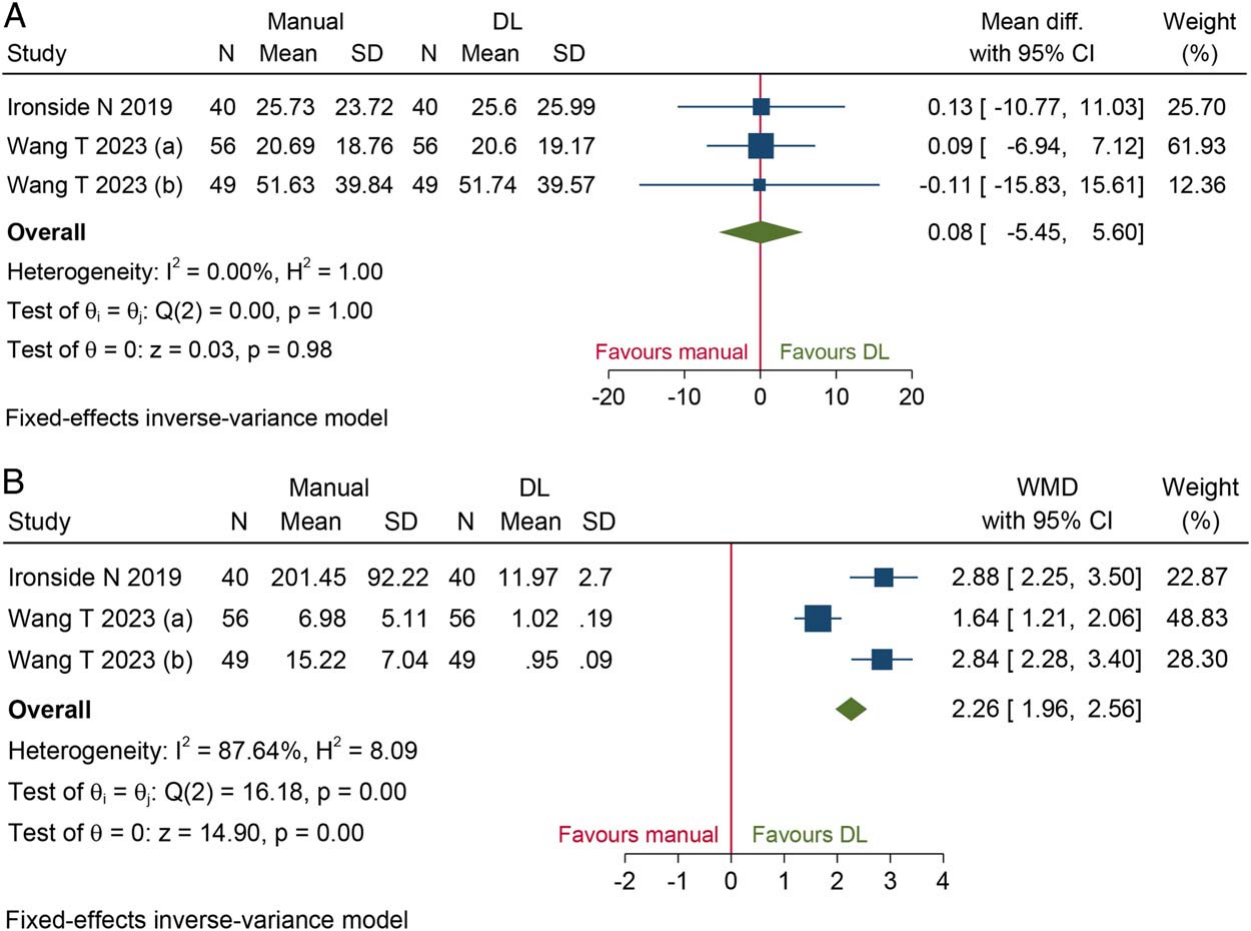
Forest plots of (A) bleeding volume; (B) process time demonstrated that there was no significant difference in bleeding volume between manual labeling and deep learning model. DL, deep learning; MD, mean difference; WMD, weight mean difference.

We compared the consistency between manual label and DL model in bleeding volume quantification. Two studies^15,40^ reported original data. Pooled results (MD 0.08, 95% CI: −5.45–5.60) suggested that regarding bleeding volume, there was no significant difference between manual label and DL model (Fig. 4A). These two studies also recorded process time. As Figure 4B presented, DL technology took less time than manual method (WMD 2.26, 95% CI: 1.96–2.56).

### Publication bias

Funnel plots and Egger test were used to estimate publication bias (Supplementary Figure 8, Supplemental Digital Content 4, http://links.lww.com/JS9/C84). Publication bias may exist in sensitivity (Egger *P*-value 0.0023), specificity (Egger *P*-value 0.0322), PPV (Egger *P*-value 0.0001), and process time (Egger *P*-value 0.0001).

## Discussion

To the best of our knowledge, this systemic review and meta-analysis was the first to make a conclusion that application of DL methods and evaluate the performance of ICH segmentation and quantification. The results showed that DL technology has comparable ability to accurately detect ICH with high sensitivity (0.89), specificity (0.91), AUROC (0.94), PPV (0.92), NPV (0.94), and precision (0.83). We also find that DL model own excellent segmenting performance with high DSC (0.84). Interestingly, with respect to the hemorrhage quantification, we did not find any significant difference between manual label and DL technology, but the latter took less time. These findings indicate that DL methods has great promise on rapid and accurate detection and segmentation of hemorrhage in acute stroke patients, and benefit for clinical procedure.

Early diagnosis of ICH is crucial for the successful management of acute stroke patients. The 2023 Guideline for the management of patients with aneurysmal subarachnoid hemorrhage proposes neurosurgeon must maintain a high level of diagnosis ability^62^. Recently, a series of tools has been developed to serve as a clinical assistant to identify ICH, such as DL model^46^, near-infrared spectroscopy (NIRS)^63^. A systematic review, conducted in 2021, indicated NIRS presented as average sensitivity 0.90 and specificity 0.77 in ICH detection^64^. However, low specificity may limit the application of NIRS. By contrast, in our analysis, the average sensitivity and specificity of DL model in ICH detection reached at 0.89 and 0.91, respectively. Furthermore, LR plot indicated DL model possess good confirmatory capabilities, which stand in line with high sensitivity characteristics of DL model. Matsoukas *et al*.^12^ collected evidence on AI-driven tools (developed by NCCT/MRI images) in detection of ICH and chronic cerebral microbleeds (CMBs) and concluded that implemented AI-driven tools in clinical practice may enhance workflow and served as a failsafe for the detection of ICH. Different from Matsoukas *et al*., we merely focused on the performance of NCCT-based DL model in ICH patients.

By providing accurate segmentation and volume quantification of different ICH subtypes, these methods can help clinicians formulate accurate diagnosis and treatment plans, especially in cases of mass effect due to hematoma. In the following discussion, we will discuss existing DL algorithms for ICH segmentation and volume quantification, and provide our insights on their strengths and weaknesses.

### Purely CNN algorithm in ICH and subtypes segmentation

As a mainstream algorithm for ICH segmentation and quantification, several studies have reported the performance of CNN-based DL algorithms in different ICH subtypes. In 2019, Patel *et al*.^49^ applied a pure 3D-CNN algorithm to construct a segmentation model of IPH, which had a Dice coefficient of 0.905 in 75 NCCTs. Unfortunately, the model has not been independently verified and its generalization cannot be known. Ironside *et al*.^40^ enrolled 300 patients with spontaneous ICH, divided into 260 training sets and 40 validation sets, and developed a fully automated segmentation algorithm using CNNs. The results showed that the DSC of the model in the training and validation sets was 0.894±0.264 and 0.905±0.254, respectively, and the quantified hematoma volume came from fully automatic and manual (R^2^=0.981; *P*<0.0001), fully automatic and semi-automatic (R^2^=0.978; The *P*<0.0001) segmentation method had a strong intergroup correlation. The results show that the CNN automatic segmentation algorithm has high accuracy in hematoma quantification, which greatly improves the efficiency, but the model is only for supratentorial IPH and lacks the verification of other subtypes and independent external test sets.

### Purely FCN in ICH and subtypes segmentation

As an upgraded version of the CNN algorithm, FCN was first proposed by Shelhamer *et al*.^65^ in 2015. Kuo *et al*.^66^ collected two large databases with a total of 4396 participants for the development, validation, and testing of the ICH segmentation model by the FCN algorithm. The results showed that the median Dice of the model in the ICH subtype was 0.75. Dhar *et al*.^11^ trained an FCN fully automated segmentation algorithm using the image data of 124 supratentorial IPH patients, which had a DSC of 0.9 in the external validation set (84 IPH patients), 0.98 compared with a manually segmented ICC, and a median bleeding volume of 18 ml, which is a lack of other bleeding subtypes, as well as the compassion of CNN and FCN algorithms in ICH segmentation and quantification.

### U-Net algorithm in ICH and subtypes segmentation

More recently, it has been reported in the literature to use the U-Net algorithm for ICH and subtypes segmentation. Xu *et al*.^67^ retrospectively collected 3000 ICH (IPH, EDH, SDH) image data and performed hematoma segmentation training by intensive U-Net frame. The experimental results show that the DSC of IPH, ETH, and SDH on the test set of the model is 0.90±0.06, 0.88±0.12, and 0.82±0.16, respectively, and the correlation coefficient of ICH volume quantification between dense U-Net and manual segmentation is 0.99. On this basis, Yu *et al*.^56^ retrospectively included 512 patients and prospectively included 50 patients with ICH (IPH, IVH, SDH, EDH) and developed a robust Unet (DR-UNet) segmentation model for hematoma volume quantification. The results show that the DR-UNet model achieves similar performance to expert clinicians in two independent test datasets containing an internal test set (DSC: 0.861±0.139) and an external test set (DSC: 0.874±0.13). The hematoma volume derived from DR-UNet was strongly correlated with the measured value of manual segmentation (*R*^*2*^: 0.9979; *P*<0.0001). In the irregularly shaped hematoma group and SDH and EDH subtypes, DR-UNet outperformed the UNet model alone in hematoma segmentation and HV measurement. Encouragingly, the average processing time for the model to process data for each NCCT scan is only 1.2 s, which is the fastest model reported to date.

### Limitations

There are several limitations should be observed in this study. Firstly, there is currently no standard data available to test all DL tools reported, making it difficult to assess the actual segmentation performance and generalization of these DL models. Secondly, the included studies were not uniform in the types of ICH segmentation, and the extracted accuracy metrics used for pooled analysis may be biased. Therefore, it is necessary and important to establish a balanced dataset to compare the performance of different DL algorithms in ICH subtypes segmentation and bleeding volume quantification. Thirdly, we attempted to search for eligible studies as much as we could, however, *P*-value of Egger test still indicated there may exist publication bias in our analysis. Therefore, the above conclusions should be interpreted with caution. Fourthly, as the nature of DL studies, we were not able to perform classical statistical comparison of measures of diagnostic accuracy between different imaging modalities.

## Conclusions

This systematic review has identified a range of DL algorithms that the performance was comparable to experienced clinicians in hemorrhage lesions identification, segmentation, and quantification but with greater efficiency and reduced cost. It is highly emphasized that multicenter randomized controlled clinical trials will be needed to validate the performance of these tools in the future, paving the way for fast and efficient decision-making during clinical procedure in patients with acute hemorrhagic stroke.

## Ethical approval

This study did not involve patients’ individual information.

## Consent

This study did not involve patients’ individual information.

## Sources of funding

This work was supported by the Natural Science Foundation of Jiangxi Province Project (No. 20224BAB216074 for TFY), Training Program for academic and technical leaders in major disciplines in Jiangxi Province - Young Talents Project (No. 20225BCJ23024 for MJW), and Graduate Student Innovation Special Fund Project of Jiangxi Province (YC2023-B081 for PH).

## Author contribution

P.H.: conceptualization, data curation, formal analysis, funding acquisition, investigation, methodology, software, supervision, validation, visualization, writing – original draft, and writing – review and editing; T.Y.: conceptualization, data curation, funding acquisition, software, supervision, writing – original draft, and writing – review and editing; B.X.: conceptualization, data curation, formal analysis, visualization, writing – original draft, and writing – review and editing; H.S.: data curation, formal analysis, investigation, methodology, resources, software, supervision, validation, and visualization; Y.S.: resources, software, validation, and visualization; Y.W.: resources, software, validation, and visualization; L.S.: data curation and formal analysis; S.L.: formal analysis, software, and supervision; M.Y.: resources, software, and supervision; Y.G.: data curation, writing – original draft, and writing – review and editing; M.W.: conceptualization, funding acquisition, project administration, writing – original draft, and writing – review and editing; X.Z.: conceptualization, project administration, writing – original draft, and writing – review and editing.

## Conflicts of interest disclosure

The authors declare that they have no conflicts of interest in this work.

## Research registration unique identifying number (UIN)

This study has been registered in PROSPERO (No. CRD42023398054).

## Guarantor

Xingen Zhu.

## Data availability statement

Data sharing not applicable as no datasets generated and/or analyzed for this study. All data relevant to the study are included in the article or uploaded as supplementary information. Additional data are not available.

## Provenance and peer review

None.

## Supplementary Material
