# Supplementary material for: Deep learning-assisted detection and segmentation of intracranial hemorrhage in noncontrast computed tomography scans of acute stroke patients: a systematic review and meta-analysis
Source: Int J Surg. 2024 Mar 15;110(6):3839–47. doi: 10.1097/JS9.0000000000001266 (PMC11175741; doi:10.1097/JS9.0000000000001266)
Supplement: Supplementary file 2 [file js9-110-3839-s002.docx]

**Identification of studies via databases and registers**

Records removed *before screening*:

Duplicate records removed (n = 89)

Records identified from:

Databases (n = 1531)

**Identification**

Records screened

(n = 1442)

Records excluded**

(n = 1333)

Reports sought for retrieval

(n = 109)

Reports not retrieved

(n = 58)

**Screening**

Reports assessed for eligibility

(n = 51)

Reports excluded:

Non-English text (n = 4)

Case/case series, review (n = 9)

Deep learning based on MRI (n = 2)

Studies included in review

(n = 36)

**Included**
