# Supplementary material for: Deep learning-assisted detection and segmentation of intracranial hemorrhage in noncontrast computed tomography scans of acute stroke patients: a systematic review and meta-analysis
Source: Int J Surg. 2024 Mar 15;110(6):3839–47. doi: 10.1097/JS9.0000000000001266 (PMC11175741; doi:10.1097/JS9.0000000000001266)
Supplement: Supplementary file 3 [file js9-110-3839-s003.docx]

AMSTAR 2: a critical appraisal tool for systematic reviews that include randomised or non- randomised studies of healthcare interventions, or both

| **1. Did the research questions and inclusion criteria for the review include the components of PICO?**   \| √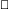 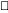  For Yes:  √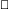 Population √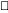 Intervention  Yes No  Optional (recommended)  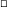 Timeframe for follow-up  √ Comparator group  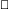 Outcome \| \| --- \|   **2. Did the report of the review contain an explicit statement that the review methods were**  **established prior to the conduct of the review and did the report justify any significant deviations from the protocol?**   \| 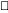 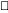 √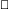  For Yes:  As for partial yes, plus the protocol should be registered and should also have specified:  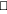 a meta-analysis/synthesis plan, if appropriate, *and*  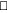 a plan for investigating causes of heterogeneity  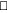 justification for any deviations from the protocol  Yes  Partial Yes No  For Partial Yes:  The authors state that they had a written protocol or guide that included ALL the following:  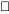 review question(s)  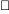 a search strategy  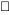 inclusion/exclusion criteria 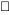 a risk of bias assessment \| \| --- \|   **3. Did the review authors explain their selection of the study designs for inclusion in the review?**   \| For Yes, the review should satisfy ONE of the following:  √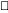 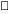  Yes No  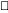 *Explanation for* including only RCTs  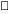 OR *Explanation for* including only NRSI  √ OR *Explanation for* including both RCTs and NRSI \| \| --- \|   **4. Did the review authors use a comprehensive literature search strategy?**   \| 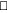 √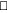 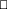  For Yes, should also have (all the following):  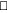 searched the reference lists / bibliographies of included  studies  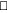 searched trial/study registries 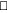 included/consulted content  experts in the field  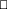 where relevant, searched for  grey literature  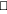 conducted search within 24  months of completion of the review  Yes  Partial Yes No  For Partial Yes (all the following):  √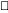 searched at least 2 databases (relevant to research question)  √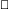 provided keyword and/or search strategy  √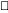 justified publication restrictions (e.g. language) \| \| --- \|   **5. Did the review authors perform study selection in duplicate?**   \| For Yes, either ONE of the following:  √ at least two reviewers independently agreed on selection of eligible studies  √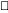 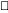  Yes No  and achieved consensus on which studies to include  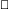 OR two reviewers selected a sample of eligible studies and achieved good  agreement (at least 80 percent), with the remainder selected by one  reviewer. \| \| --- \| |
| --- | --- | --- | --- | --- | --- |

AMSTAR 2: a critical appraisal tool for systematic reviews that include randomised or non- randomised studies of healthcare interventions, or both

| **6. Did the review authors perform data extraction in duplicate?**   \| For Yes, either ONE of the following:  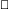 at least two reviewers achieved consensus on which data to extract from  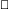 √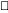  Yes No  included studies  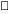 OR two reviewers extracted data from a sample of eligible studies and  achieved good agreement (at least 80 percent), with the remainder  extracted by one reviewer. \| \| --- \|   **7. Did the review authors provide a list of excluded studies and justify the exclusions?**   \| 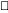 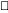 √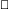  For Partial Yes:  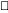 provided a list of all potentially relevant studies that were read in full-text form but excluded from the review  Yes  Partial Yes No  For Yes, must also have:  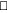 Justified the exclusion from the review of each potentially relevant study \| \| --- \|   **8. Did the review authors describe the included studies in adequate detail?**  For Yes, should also have ALL the following:  √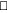 described population in detail √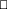 described intervention in  detail (including doses where relevant)  √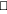 described comparator in detail (including doses where  relevant)  √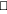 described study’s setting  √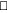 timeframe for follow-up   \| √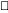 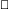 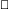  Yes  Partial Yes No  For Partial Yes (ALL the following):   \| 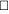  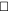  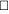  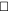  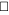 \| described described described described described \| populations  interventions  comparators  outcomes  research designs \| \| --- \| --- \| --- \| \| \| --- \| --- \| --- \| --- \|   **9. Did the review authors use a satisfactory technique for assessing the risk of bias (RoB) in individual studies that were included in the review?**   \| **RCTs**  For Partial Yes, must have assessed RoB  For Yes, must also have assessed RoB from:  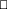 allocation sequence that was not truly random, *and*  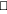 selection of the reported result from among multiple  measurements or analyses of a specified outcome  from  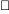 unconcealed allocation, *and*  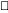 Yes  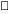 Partial Yes 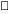 No  √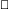 Includes only NRSI  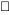 lack of blinding of patients and  assessors when assessing  outcomes (unnecessary for  objective outcomes such as all-  cause mortality) \| \| --- \| \| **NRSI**  For Partial Yes, must have assessed  For Yes, must also have assessed RoB: √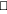 methods used to ascertain  exposures and outcomes, *and*  √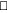 selection of the reported result from among multiple  √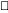 Yes  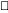 Partial Yes 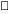 No  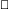 Includes only RCTs  RoB:  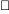 from confounding, *and*  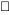 from selection bias  measurements or analyses of a  specified outcome \|   **10. Did the review authors report on the sources of funding for the studies included in the review?**   \|  \| For Yes \|  \|  \|  \| \| --- \| --- \| --- \| --- \| --- \| \|  \| √ \| Must have reported on the sources of funding for individual studies included \| √ \| Yes \| \|  \|  \| in the review. Note: Reporting that the reviewers looked for this information \| 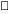 \| No \| \|  \|  \| but it was not reported by study authors also qualifies \|  \|  \| |
| --- | --- | --- | --- | --- | --- | --- | --- | --- | --- | --- | --- | --- | --- | --- | --- | --- | --- | --- | --- | --- | --- | --- | --- | --- | --- | --- | --- | --- |

AMSTAR 2: a critical appraisal tool for systematic reviews that include randomised or non- randomised studies of healthcare interventions, or both

| **11. If meta-analysis was performed did the review authors use appropriate methods for statistical combination of results?**   \| **RCTs**  For Yes:  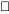 The authors justified combining the data in a meta-analysis  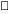 Yes  √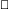 No  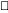 No meta-analysis conducted  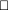 AND they used an appropriate weighted technique to combine  study results and adjusted for heterogeneity if present.  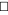 AND investigated the causes of any heterogeneity \| \| --- \| \| **For NRSI**  For Yes:  √ The authors justified combining the data in a meta-analysis  √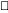 Yes  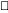 No  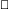 No meta-analysis conducted  √ AND they used an appropriate weighted technique to combine  study results, adjusting for heterogeneity if present  √ AND they statistically combined effect estimates from NRSI that  were adjusted for confounding, rather than combining raw data,  or justified combining raw data when adjusted effect estimates  were not available  √ AND they reported separate summary estimates for RCTs and  NRSI separately when both were included in the review \|   **12. If meta-analysis was performed, did the review authors assess the potential impact of RoB in individual studies on the results of the meta-analysis or other evidence synthesis?**   \| For Yes:  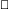 OR, if the pooled estimate was based on RCTs and/or NRSI at variable  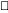 included only low risk of bias RCTs  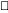 Yes  √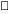 No  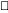 No meta-analysis conducted  RoB, the authors performed analyses to investigate possible impact of  RoB on summary estimates of effect. \| \| --- \|   **13. Did the review authors account for RoB in individual studies when interpreting/ discussing the results of the review?**   \| For Yes:  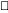 included only low risk of bias RCTs  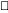 √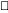  Yes No  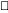 OR, if RCTs with moderate or high RoB, or NRSI were included the  review provided a discussion of the likely impact of RoB on the results \| \| --- \|   **14. Did the review authors provide a satisfactory explanation for, and discussion of, any heterogeneity observed in the results of the review?**   \| For Yes:  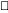 There was no significant heterogeneity in the results  √ OR if heterogeneity was present the authors performed an investigation of  √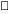 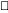  Yes No  sources of any heterogeneity in the results and discussed the impact of this  on the results of the review \| \| --- \|   **15. If they performed quantitative synthesis did the review authors carry out an adequate**  **investigation of publication bias (small study bias) and discuss its likely impact on the results of the review?**   \| For Yes:  √ performed graphical or statistical tests for publication bias and discussed  √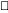 Yes  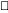 No  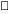 No meta-analysis conducted  the likelihood and magnitude of impact of publication bias \| \| --- \| |
| --- | --- | --- | --- | --- | --- | --- |

AMSTAR 2: a critical appraisal tool for systematic reviews that include randomised or non- randomised studies of healthcare interventions, or both

| **16. Did the review authors report any potential sources of conflict of interest, including any funding they received for conducting the review?** | | | |
| --- | --- | --- | --- |
| For Yes:  √ The authors reported no competing interests OR  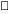 The authors described their funding sources and how they managed | √ 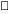 | Yes  No |  |
| potential conflicts of interest  | | |  |

**To cite this tool:** Shea BJ, Reeves BC, Wells G, Thuku M, Hamel C, Moran J, Moher D, Tugwell P, Welch V, Kristjansson E, Henry DA. AMSTAR 2: a critical appraisal tool for systematic reviews that include randomised or non-randomised studies of healthcare interventions, or both. BMJ. 2017 Sep 21;358:j4008.
