# Supplementary material for: Deep learning-assisted detection and segmentation of intracranial hemorrhage in noncontrast computed tomography scans of acute stroke patients: a systematic review and meta-analysis
Source: Int J Surg. 2024 Mar 15;110(6):3839–47. doi: 10.1097/JS9.0000000000001266 (PMC11175741; doi:10.1097/JS9.0000000000001266)
Supplement: Supplementary file 4 [file js9-110-3839-s004.docx]

**Supplemental material**

**Deep learning-assisted detection and segmentation of intracranial hemorrhage in non-contrast computed tomography scans of acute stroke patients: a systematic review and meta-analysis**

**SDC, Table 1.** Characteristics of the included studies

**SDC, Table 2**. Summary of quality assessment as regards the individual studies

**SDC, Figure 1.** Pooled results of sensitivity

**SDC, Figure 2.** Pooled results of specificity

**SDC, Figure 3.** Pooled results of AUROC

**SDC, Figure 4.** Pooled results of positive predictive value

**SDC, Figure 5.** Pooled results of negative predictive value

**SDC, Figure 6.** Pooled results of precision

**SDC, Figure 7.** Pooled results of dice similarity coefficient

**SDC, Figure 8.** Funnel plot for sensitivity, specificity, AUROC, PPV, NPV, precision, DCS, Volume of bleeding, and process time

**SDC, Figure 9.** Deeks’ funnel plot asymmetry test

**SDC, Tabel 1.** Characteristics of the included studies.

| **Study (year)** | **Country** | **ICH classification** | **Model** | **Population** | **Test set** | **Type of internal validation** | **External validation** | **Deep learning task** | **Ground truth definer** | **Performance measurements** |
| --- | --- | --- | --- | --- | --- | --- | --- | --- | --- | --- |
| Abramova V 2021 | Spain | IPH | 3D SE U-Net | Images | 15 | 5-fold cross-validation | No | Segmentation | Expert radiologists | DSC |
| Alis D 2022 | Turkey | IPH, IVH, SAH, SDH, EDH | Joint CNN-RNN | Scans | 452 | 5-fold cross-validation | No | Detection | Neuroradiologists and expert radiologists | Sensitivity, Specificity, Precision, AUROC |
| Arab A 2020 | Canada | IPH, IVH | CNN-DS | Patients | 10 | Random split | No | Segmentation and volume quantification | Expert radiologists | DSC, Precision, Recall |
| Arbabshirani MR 2018 | USA | NR | CNN | Patients | 1. 9499 (dataset) 2. 347 (clinical implementation) | Random split | Yes | Detecting | Neuroradiologist | Sensitivity, Specificity, AUROC |
| Chang PD 2018 | USA | IPH, EDH, SDH, SAH | Mask R-CNN | Patients | 682 | 5-fold cross-validation | No | Detection and volume quantification, segmentation | Board-certified radiologist | Sensitivity, Specificity,  PPV, Precision |
| Chilamkurthy S 2018 | India | IPH, IVH, SDH, EDH, SAH | CNN | Scans | 1. 21095 (Qure25k dataset) 2. 491 (CQ500 dataset) | Random split | Yes | Detection | Original clinical radiology report and radiologists | Sensitivity, Specificity, AUROC |
| Colasurdo M 2023 | USA | SDH | CNN | Scans | 340 | Random split | Yes | Detection and volume quantification, segmentation | Specialized clinical annotator | Sensitivity, Specificity, PPV, NPV |
| Coorens NA 2023 | USA | IPH | Masked loss U-Net | Patients | 22 | Random split | No | Detection, segmentation | Expert radiologists | Sensitivity, Specificity, DSC |
| Cao H 2023 | Germany | IVH | 3D CNN | Patients | 920 | Random split | No | Detection, segmentation volume, quantification | Expert radiologists | Sensitivity, PPV, DCS |
| Farzaneh N 2020 | USA | SDH | Deep learning with 3D post-processing | Patients | NR | 10 folds for cross-validation | No | Detection and volume quantification, segmentation | Neuroradiologists | DSC, Precision, Recall |
| Ginat DT 2020 | USA | IPH, IVH, SDH, EDH, SAH, EAH not otherwise specified, Indeterminate | CNN | Patients | NR | NR | No | Detection | Depending on hemorrhage type and size and included both weak and strong labeling schema | Sensitivity, Specificity, PPV, NPV |
| Guo Y 2022 | China | IPH, IVH, SDH, EDH, SAH | RoLo: Annotator-free and weakly supervised deep-learning system | Scans | 1. 2357 (retrospective) 2. 650 (prospective) 3. 1525 (cross-centre) 4. 1484 (cross-equipment) 5. 491 (CQ500) | Random split | Yes | Detection | Expert radiologists | Sensitivity, Specificity, PPV, NPV, AUROC |
| Heit JJ 2021 | USA | IPH, SDH, IVH, EAH | hybrid 2D–3D CNN | Patients | 62 | Random split | No | Detection and volume quantification, segmentation | Neuroradiology | Sensitivity, Specificity, PPV, NPV |
| Hu P 2023 | China | SAH, IPH, IVH | Hybrid 2D/3D UNet | Patients | 1. 186 (Nanchang) 2. 101 (Wuhan) 3. 179 (Chongqing) 4. 144 (Hangzhou) | Random split | Yes | Detection and volume quantification, segmentation | Neurosurgeons and radiologists | DSC, Precision, Recall |
| Hua Y 2022 | China | IVH | 1. UNet 2. SA- UNet 3. UNet2B 4. SA- UNet2B 5. Att UNet 6. SA-Att UNet | Patients | 89 | 5-fold cross-validation | No | Detection and segmentation | Expert radiologists | Sensitivity, Specificity, DSC |
| Ironside N 2019 | USA | IVH, SAH | U-Net CNN | Patients | 40 | 5-fold cross-validation | No | Detection and volume quantification, segmentation | NR | DSC, Process time  Volume of bleeding |
| Jiang X 2022 | China | NR | 2D/3D sICHNet | Images | 10 | 10-fold cross-validation | Yes | Segmentation | Expert radiologists | DSC |
| Kellogg RT 2020 | USA | SDH | 3D CNN | Patients | 26 | Hold- out method | No | Segmentation | Neuroradiologists | DSC |
| KN BP 2012 | USA | NR | 1. Modified threshold 2. Modified FCM 3. Modified NCut | Scans | 41 | NR | No | Detection and volume quantification, segmentation | Trained CT reader and neurologist | Sensitivity, Specificity, DSC |
| Kok YE 2022 | England | NR | Une, DeepLabv3+ | Patients | 174 | 5-fold cross-validation | No | Detection, volume quantification, segmentation | Neurologist, stroke physician, radiographer | DSC |
| Kuang Z 2020 | China | NR | Ψ-Net (based on U-Net and follows the encoder-decoder framework) | Scans | 1. 30 (spontaneous) 2. 13 (traumatic) | NR | No | Detection, volume quantification, segmentation | Expert radiologists | Sensitivity, Specificity, DCS |
| Lee H 2019 | USA | IPH, IVH, SDH, EDH, SDH | Explainable deep-learning system | Patients | 200 | Random split | Yes | Detection | Expert radiologists | Sensitivity, Specificity, AUROC |
| McLouth J 2021 | USA | IPH, IVH, EDH, SDH, SAH | CINA ® v1.0 device (Avicenna.ai, La Ciotat, France) | Patients | 814 | Random split | Yes | Detection | Board-certified neuroradiologists | Sensitivity, Specificity |
| Nijiati M 2022 | China | IPH, IVH, EDH, SDH, SAH | Symmetric Transformer network | Patients | 200 | Hold- out method | No | Segmentation | Expert radiologists | Sensitivity, Specificity, DCS |
| Patel A 2019 | Netherlands | NR | 3D CNN | Patients | 30 | NR | No | Segmentation | Expert neuroradiologists | DCS |
| Phaphuangwittayakul A 2022 | Thailand | IPH, IVH, EDH, SDH, SAH | A double-branch deep neural network | Patients | 321 | Hold- out method | Yes | Detection and volume quantification, segmentation | Expert neuroradiologists | DCS |
| Rava RA 2021 | USA | IPH, IVH, SDH, SAH, EDH | ^AUTO^Stroke platform | Patients | 302 | NR | Yes | Detection and segmentation | Expert neuroradiologists | Sensitivity, Specificity, PPV, NPV |
| Schmitt N 2022 | Germany | IPH | CNN | Patients | 160 | NR | NR | Detection and volume quantification, segmentation | Expert neuroradiologists | Sensitivity, Specificity, AUROC |
| Thanellas A 2023 | Finland | SAH | 2D 5-level U-Net-type architecture | Patients | 1989 | NR | NR | Detection, and segmentation | According to axial MPR planes | Sensitivity, Specificity, Precision |
| Tong G 2023 | China | IPH, IVH | 3D MBA U-Net | Patients | 103 | Random split | Yes | Detection, and segmentation, volume quantification | Expert neuroradiologists | Sensitivity, Specificity, Precision, DSC |
| Voter AF 2021 | USA | IPH, IVH, SDH, SAH, EDH, EAH | MBA-UNet | Patients | 3605 | NA | NA | Detection | Expert neuroradiologists | Sensitivity, Specificity, PPV, NPV |
| Wang X 2021 | China | IPH, IVH, SDH, SAH, EDH | Mimics the interpretation process of radiologists, and combines a 2D CNN model and two sequence models | Scans | 2214 | 5-fold cross-validation | Yes | Detection and classification | Expert radiologists | Sensitivity, Specificity, Precision |
| Wang T 2023 | China | IPH, IVH, SDH, SAH, EDH | Dense-Net and U-Net | Patients | 1. 56 (ICH without IVH) 2. 49 (ICH with IVH) | NA | NA | Detection, and segmentation, volume quantification | Computed tomography (CT)-based planimetry | Process time  Volume of bleeding |
| Yu N 2022 | China | NR | Dimension reduction UNet | Images | 854 | Random split | Yes | Detection and volume quantification, segmentation | Board-certified neuroradiologist | Sensitivity, Specificity, Precision, DSC |
| Zhao X 2021 | China | IVH | nnU-Net | Patients | 80 | Random split | No | Detection and volume quantification, segmentation | Expert radiologists | Sensitivity, PPV, DSC |
| Zhou Z 2022 | China | NR | Region-growing algorithm based on watershed preprocessing | Patients | 55 | NR | NR | Detection and volume quantification, segmentation | Experienced clinicians | Sensitivity, PPV, DSC |

CNN-RNN, convolutional neural networks-recurrent neural networks; 3D SE U-Net, 3D U-Net with squeeze-and-excitation; CNN-DS, convolutional neural networks with deep supervision; NA, not reported; ICH, intracranial hemorrhage; IVH, intraventricular hemorrhage; IPH, intra-parenchymal hemorrhage; SAH, subarachnoid hemorrhage; SDH, subdural hemorrhage; EDH, epidural hemorrhage; DSC, dice similarity coefficient; AUROC, Area under receiver operation characteristic curve; PPV, positive predictive value; NPV, negative predictive value; EAH, extra-axial hematoma; NA, not application.

**SDC, Tabel 2.** Summary of quality assessment as regards the individual studies.

| **Study (year)** | **RISK OF BIAS** | | | | **APPLICABILITY CONCERNS** | | |
| --- | --- | --- | --- | --- | --- | --- | --- |
|  | **PATIENT**  **SELECTION** | **INDEX TEST** | **REFERENCE**  **STANDARD** | **FLOW AND**  **TIMING** | **PATIENT**  **SELECTION** | **INDEX TEST** | **REFERENCE**  **STANDARD** |
| Abramova V 2021 | 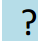 | 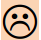 | 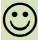 | 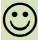 | 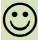 | 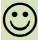 | 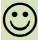 |
| Alis D 2022 | 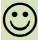 | 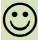 | 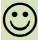 | 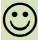 | 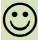 | 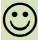 | 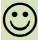 |
| Arab A 2020 | 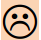 | 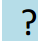 | 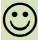 | 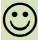 | 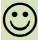 | 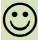 | 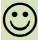 |
| Arbabshirani MR 2018 | 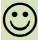 | 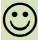 | 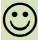 | 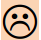 | 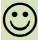 | 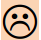 | 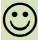 |
| Cao H 2023 | 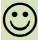 | 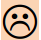 | 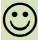 | 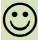 | 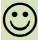 | 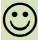 | 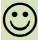 |
| Chang PD 2018 | 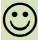 | 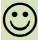 | 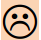 | 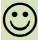 | 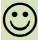 | 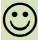 | 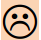 |
| Chilamkurthy S 2018 | 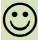 | 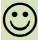 | 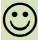 | 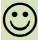 | 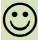 | 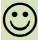 | 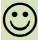 |
| Colasurdo M 2023 | 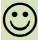 | 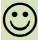 | 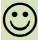 | 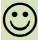 | 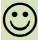 | 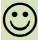 | 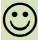 |
| Coorens NA 2023 | 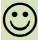 | 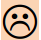 | 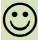 | 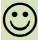 | 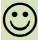 | 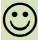 | 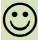 |
| Farzaneh N 2020 | 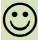 | 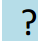 | 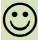 | 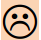 | 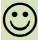 | 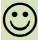 | 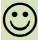 |
| Ginat DT 2020 | 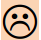 | 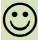 | 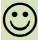 | 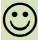 | 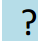 | 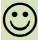 | 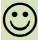 |
| Guo Y 2022 | 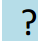 | 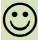 | 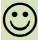 | 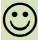 | 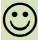 | 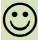 | 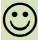 |
| Heit JJ 2021 | 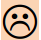 | 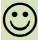 | 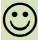 | 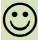 | 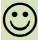 | 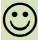 | 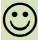 |
| Hu P 2023 | 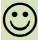 | 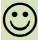 | 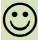 | 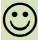 | 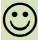 | 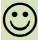 | 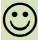 |
| Hua Y 2022 | 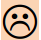 | 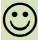 |  |  |  |  |  |
| Ironside N 2019 |  |  |  |  |  |  |  |
| Jiang X 2022 |  |  |  |  |  |  |  |
| Kellogg RT 2020 |  |  |  |  |  |  |  |
| KN BP 2012 |  |  |  |  |  |  |  |
| Kok YE 2022 |  |  |  |  |  |  |  |
| Kuang Z 2020 |  |  |  |  |  |  |  |
| Lee H 2019 |  |  |  |  |  |  |  |
| McLouth J 2021 |  |  |  |  |  |  |  |
| Nijiati M 2022 |  |  |  |  |  |  |  |
| Patel A 2019 |  |  |  |  |  |  |  |
| Phaphuangwittayakul A 2022 |  |  |  |  |  |  |  |
| Rava RA 2021 |  |  |  |  |  |  |  |
| Schmitt N 2022 |  |  |  |  |  |  |  |
| Thanellas A 2023 |  |  |  |  |  |  |  |
| Tong G 2023 |  |  |  |  |  |  |  |
| Voter AF 2021 |  |  |  |  |  |  |  |
| Wang X 2021 |  |  |  |  |  |  |  |
| Wang T 2023 |  |  |  |  |  |  |  |
| Yu N 2022 |  |  |  |  |  |  |  |
| Zhao X 2021 |  |  |  |  |  |  |  |
| Zhou Z 2022 |  |  |  |  |  |  |  |

**Low Risk** **High Risk** **Unclear Risk**

**SDC, Figure 1.** Pooled results of sensitivity

**SDC, Figure 2.** Pooled results of specificity

**SDC, Figure 3.** Pooled results of AUROC

**SDC, Figure 4.** Pooled results of positive predictive value

**SDC, Figure 5.** Pooled results of negative predictive value

**SDC, Figure 6.** Pooled results of precision

**SDC, Figure 7.** Pooled results of dice similarity coefficient

**SDC, Figure 8.** Funnel plot for sensitivity, specificity, AUROC, PPV, NPV, precision, DCS, volume of bleeding, and process time

(A) sensitivity; (B) specificity; (C) AUROC; (D) PPV; (E) NPV; (F) precision; (G) DCS; (H) volume of bleeding; (I) process time.

**SDC, Figure 9.** Deeks’ funnel plot asymmetry test
